# Astrocytic endothelin-1 overexpression promotes neural progenitor cells proliferation and differentiation into astrocytes via the Jak2/Stat3 pathway after stroke

**DOI:** 10.1186/s12974-019-1597-y

**Published:** 2019-11-16

**Authors:** Xiao Cheng, Patrick K. K. Yeung, Ke Zhong, Prince L. M. Zilundu, Lihua Zhou, Sookja K. Chung

**Affiliations:** 1 Department of Neurology, Guangdong Provincial Hospital of Traditional Chinese Medicine, 111 Dade Road, Guangzhou, 510120 China; 2 Faculty of Medicine, Macau University of Science and Technology, Macau, China; 3 School of Biomedical Sciences, The University of Hong Kong, HKSAR, China; 4 The Second Affiliated Hospital of Guangzhou University of Chinese Medicine, 12 Jichang Road, Guangzhou, 510405 China; 5 Guangdong Provincial Chinese Emergency Key Laboratory, Guangzhou, 510120 China; 6 State Key Laboratory of Dampness Syndrome of Traditional Chinese Medicine, Guangzhou, 510120 China; 7 Department of Anatomy, Zhong Shan School of Medicine, Sun Yat-Sen University, Guangdong Province, Guangzhou, China

**Keywords:** Endothelin-1, Neural progenitor cells, Astrocyte, Jak2/Stat3, Transient middle cerebral artery occlusion

## Abstract

**Background:**

Endothelin-1 (ET-1) is synthesized and upregulated in astrocytes under stroke. We previously demonstrated that transgenic mice over-expressing astrocytic ET-1 (GET-1) displayed more severe neurological deficits characterized by a larger infarct after transient middle cerebral artery occlusion (tMCAO). ET-1 is a known vasoconstrictor, mitogenic, and a survival factor. However, it is unclear whether the observed severe brain damage in GET-1 mice post stroke is due to ET-1 dysregulation of neurogenesis by altering the stem cell niche.

**Methods:**

Non-transgenic (Ntg) and GET-1 mice were subjected to tMCAO with 1 h occlusion followed by long-term reperfusion (from day 1 to day 28). Neurological function was assessed using a four-point scale method. Infarct area and volume were determined by 2,3,5-triphenyltetra-zolium chloride staining. Neural stem cell (NSC) proliferation and migration in subventricular zone (SVZ) were evaluated by immunofluorescence double labeling of bromodeoxyuridine (BrdU), Ki67 and Sox2, Nestin, and Doublecortin (DCX). NSC differentiation in SVZ was evaluated using the following immunofluorescence double immunostaining: BrdU and neuron-specific nuclear protein (NeuN), BrdU and glial fibrillary acidic protein (GFAP). Phospho-Stat3 (p-Stat3) expression detected by Western-blot and immunofluorescence staining.

**Results:**

GET-1 mice displayed a more severe neurological deficit and larger infarct area after tMCAO injury. There was a significant increase of BrdU-labeled progenitor cell proliferation, which co-expressed with GFAP, at SVZ in the ipsilateral side of the GET-1 brain at 28 days after tMCAO. p-Stat3 expression was increased in both Ntg and GET-1 mice in the ischemia brain at 7 days after tMCAO. p-Stat3 expression was significantly upregulated in the ipsilateral side in the GET-1 brain than that in the Ntg brain at 7 days after tMCAO. Furthermore, GET-1 mice treated with AG490 (a JAK2/Stat3 inhibitor) sh owed a significant reduction in neurological deficit along with reduced infarct area and dwarfed astrocytic differentiation in the ipsilateral brain after tMCAO.

**Conclusions:**

The data indicate that astrocytic endothelin-1 overexpression promotes progenitor stem cell proliferation and astr ocytic differentiation via the Jak2/Stat3 pathway.

## Introduction

Endothelin (ET)-1 is a 21-amino acid, potent vasoconstrictor originally isolated from culture media of porcine aortic endothelial cells [1]. It is expressed in several neuronal types and involved in central autonomic control of cardiorespiratory functions [2]. In addition to its cardiovascular effects, ET-1 is involved in embryonic development [3, 4], prostate cell growth [5] carcinogenesis [6], gastrointestinal [7], and endocrine functions [8]. ET-1 is also implicated in numerous neurodegenerative diseases including stroke [9]. Previous clinical studies have shown that the level of ET-1 is significantly elevated in cerebrospinal fluid and plasma of stroke patients [10, 11]. Similar results have been reported as well in rat brain tissue following experimental cerebral ischemia [12, 13]. These results suggest a critical ET-1 role in cerebral ischemic injury pathogenesis.

Astrocytes are the major cell type responsible for ET-1 production in the brain [11, 14, 15]. In animal studies, it has been demonstrated that there is an increased ET-like immunoreactivity in the astrocyte-like cells after ischemic brain damage [2, 16]. However, it is still unclear whether the induction of astrocytic ET-1-like activity is neuroprotective or neurodegenerative following cerebral ischemia. Utilizing the astrocytic ET-1 overexpressing mice (GET-1), we previously showed that astrocytic ET-1 overexpression led to more severe ischemic brain injury [9]. In this study, we also found that the severity of the ischemic brain injury was associated with ET-1’s deleterious effects on water homeostasis, cerebral edema, and blood-brain barrier integrity. In another study, we also demonstrated that astrocytic ET-1 disrupts cognitive function as well as mediates Alzheimer’s disease and mild ischemic stroke-associated neurodegeneration [17].

ET-1 acts on astrocytes in an autocrine manner, resulting in increased cell proliferation [18–21]. An increasing body of evidence indicates that astrocytes contribute to the governance as well as fine-tuning of stem and progenitor cell production during brain development [22]. For instance, results from recent studies have demonstrated that the substances secreted by astrocytes play crucial functions in neural stem cell (NSC) fate determination [23, 24]. However, the signaling pathways and mechanisms involved in cell fate determination are yet to be elucidated. As a mitogenic factor [22], it is unclear whether such effects of astrocytic ET-1 contributes to neurogenesis or astrogenesis following cerebral ischemia.

The sub-ventricular zone (SVZ) of the lateral ventricle and the subgranular zone (SGZ) of the hippocampal dentate gyrus’ NSCs are capable of producing new neurons in the adult brain [25]. These NSCs retain the capacity to self-renew and possibly differentiate into neurons, astrocytes, or oligodendrocytes [26]. Normally, the NSCs differentiate into neural progenitor cells that migrate to other brain areas where they generate functional neurons for brain repair [25, 27]. The NSCs generated in the SVZ migrate through the rostro-migratory stream to the olfactory bulb, where they differentiate into local interneurons [28]. Several factors have previously been shown to influence NSC proliferation and neurogenesis [29]. However, the molecular mechanisms and the signaling pathways that regulate the proliferation and differentiation of NSCs as well as how those signaling molecules affect NSC neurogenesis under pathological conditions have not been well-studied.

In addition to the intrinsic properties of neural stem cells, recent studies also demonstrated that the local microenvironment or “niche,” consisting of components such as growth factors, cytokines, and cell–cell contact, plays key roles in the neural stem cell fate determination [24, 30, 31]. It is also well documented that astrocytes constitute a major component of neural microenvironment in the central nervous system (CNS) [32]. Beyond providing nutrition, support, and protection to neurons under physiological conditions, astrocytes also play crucial roles in the CNS pathologies. For instance, there is mounting evidence which support the view that CNS injuries or diseases not only elicit a characteristic inflammatory reaction, but also enhance reactive proliferation and differentiation of NSCs [31, 33]. Furthermore, astrocytes, being one of the key players mediating the inflammatory response, are markedly activated in various CNS diseases where they participate in neuronal survival, maturation, and neurogenesis or astrogenesis [23, 34]. These findings suggest that astrocytes may affect proliferation and differentiation of NSCs in the injured CNS.

In light of the above, it is important to investigate and understand the role of astrocytic ET-1 and its propensity to stimulate neurogenesis or astrogenesis following a cerebrovascular accident. Such knowledge would help expose potential drug targets for the treatment of stroke. In the present study, we investigated the effects of astrocytic ET-1 on neural progenitor cell proliferation, migration, and differentiation in the adult mouse SVZ after stroke.

## Materials and methods

### Animal husbandry

All procedures in this study were carried out in accordance with the recommendations in the Guide for the Care and Use of Laboratory Animals of the National Institutes of Health (USA, 2011). GET-1 transgenic mice and non-transgenic (Ntg) mice were generated as previously described [9]. The protocol of this study was approved by the Committee on the Use of Live Animals in Teaching and Research of the Hong Kong University. All surgical procedures were performed under anesthesia, and all efforts were made to minimize suffering. Age- and weight-matched Ntg and GET-1 male littermates were used in all experiments. The mice were kept at 21 °C ± 1 °C, 55% ± 10% humidity, in a 12-h light-dark cycle housing, with free access to food and water. Adult male Ntg and GET-1 mice (age, 2–3 months; weight, 24–28 g) were obtained from the Laboratory Animal Center of The University of Hong Kong.

### Antibodies and reagents

Bromodeoxyuridine (BrdU) was purchased from Sigma-Aldrich (St. Louis, MO, USA). AG490 was purchased from Calbiochem (San Diego, CA, USA). The following primary antibodies were used in these experiments: rat monoclonal anti-BrdU (Abcam, ab6326, 1:500), mouse anti-Sox2 (Santa Cruz, sc365823, 1:200), rabbit anti-Ki67 (Millipore, AB9260, 1:200), mouse anti-nestin (Abcam, ab6142, 1:200), rabbit anti-doublecortin (DCX) (Abcam, ab18723, 1:400), rabbit anti-glial fibrillary acidic protein (GFAP) (DAKO, 1:1500), rabbit anti-NeuN (Abcam, ab177487, 1:500), rabbit anti-Phospho-Stat3 (Tyr705) (Cell Signaling Technology #9145, 1:2000), and mouse anti-GAPDH (Abcam, ab8245, 1:10000). All secondary antibodies for immunofluorescent study were Alexa Fluor antibodies from Thermo Fisher Scientific (Waltham, MA, USA), namely goat anti-rat 488, goat anti-mouse 568, and goat anti-rabbit TRITC (T2769). All secondary antibodies for Western-blot were ECL™ anti-rabbit and anti-mouse IgG from GE Healthcare (UK) Company.

### Drug administration and experimental protocol

Information about drug administration and experimental protocol is listed in Additional file 1: Figure S1. To identify newly synthesized DNA after ischemic brain injury, BrdU (50 mg/kg) dissolved in saline was given intraperitoneally (ip) twice daily at 8 h intervals for five consecutive days, starting 24 h after transient middle cerebral artery occlusion (tMCAO) (both groups, each *n* = 5). In another control line for each group (GET-1 vs Ntg, *n* = 5), the Jak2/Stat3 inhibitor AG490 (3 mg/kg, intraperitoneal injection) or dilution vehicle 0.1% DMSO were administrated for 5 times: 30 min before surgery, 1st, 2nd, 4th, and 6th day after surgery.

To explore the effects of astrocytic endothelin-1 overexpression on neural progenitor cell proliferation and differentiation, mice were randomly divided into two groups (Additional file 1: Figure S1A): GET-1 and Ntg group. Neurological function assessment was performed at 1 day, 7 days, and 28 days post-injury; cerebral infarct area and volume were assessed at 7 days post-injury; p-Stat3 expression was determined at 7 days by Western blot. The proliferation and migration of neural progenitor cells were measured by using BrdU, Ki-67, Sox2, and Nestin. DCX immunofluorescence double-labeling in the brain at 7 days post-injury. Neural progenitor cells differentiation was evaluated using BrdU, NeuN, and GFAP immunofluorescence double-labeling in the brain at 28 days post-injury. The Jak2/Stat3 inhibitor AG490 was used in order to investigate the possible role of the Jak2/Stat3 signaling pathway in the neural progenitor cell proliferation and astrocyte-differentiation induction and ultimately the functional outcome in mice after tMCAO injury. Herein, mice were randomly divided into four groups (Additional file 1: Figure S1 B): Ntg + AG490 group, Ntg + vehicle group, GET-1 + AG490 group, and GET-1 + vehicle group; and the detection index and time-point is the same as that of the first batch.

### The transient middle cerebral artery occlusion model (tMCAO)

Transient focal ischemia was induced using an intraluminal technique to occlude the right middle cerebral artery as previously described [9]. Briefly, all mice were individually anesthetized with gas anesthesia (2% halothane in 70% N_2_O/30% O_2_ for induction and 1% halothane in 70% N_2_O/30% O_2_ for maintenance), a nylon monofilament (Johnson & Johnson, Brussels, Belgium) coated with impression material (3 mol/L Dental Products, St. Paul, MN, USA) was inserted into the right internal carotid artery to block the origin of right middle cerebral artery. Regional cerebral blood flow was monitored during the whole surgical procedure to confirm appropriate suture placement (Additional file 5: Figure S5). After an hour, the filament was pulled out to allow for reperfusion and the mouse was kept in an intensive care system (ThermoCare Inc., Incline Village, NV, USA) for 4 h before being returned to its long-term care cage.

### Neurological score assessment

Neurological assessments were performed at 1, 7, and 28 days after the onset of tMCAO in a double-blinded fashion. The neurological deficits were scored on a 4-point scale: (0) no observable neurological deficits (normal), (1) failure to extend left forepaw fully (mild), (2) circling to contralateral side (moderate), and (3) loss of walking and righting reflex (severe). The person who performed the neurological score assessments was blinded to the genotypes and treatments of the mice.

### Measurement of infarct size and volume

The brains from GET-1 and Ntg (*n* = 5, each) were collected after tMCAO and 5 two-mm-thick coronal sections were obtained. The brain slices were stained with 2% 2,3,5-triphenyltetrazolium chloride (TTC, Sigma, St. Louis, MO, U.S.A.) in the dark at 37 °C for 10–15 min. TTC reacts with intact mitochondrial respiratory enzyme to generate a bright red color that contrasts with the pale color of the infarction. The posterior surface of each slice was photographed and analyzed by an image analysis software program (Sigma Scan Pro5.0. Statistic Package for the Social Sciences: SPSS, Inc.). Infarct area and volume were calculated using an indirect method and presented as the percentage of infarct area of the contralateral hemisphere to eliminate the contribution of edema to the ischemic lesion as previously described [9].

### Double immunofluorescence labeling

For double immunofluorescent staining, the brains were fixed with 10% formalin, dehydrated serially with increasing percentage of ethanol and finally with chloroform incubation, and embedded in paraffin. Seven-micrometer-thick coronal sections were cut using microtome (Microm International GmbH, Walldorf, Germany). After antigen retrieval by hot citrate buffer (pH = 6), deparaffinized sections were incubated with blocking reagent for 1 h. Brain sections from each mice group were incubated with the following primary antibodies: rat monoclonal anti-BrdU, mouse anti-Sox2, rabbit anti-Ki67, mouse anti-nestin, rabbit anti-doublecortin, rabbit anti-GFAP, rabbit anti-NeuN, and rabbit anti-Phospho-Stat3 (Tyr705), at 4 °C overnight. The sections were then incubated with secondary antibodies accordingly for 2 h at room temperature after a through wash. The secondary antibodies used were Alexa Fluor antibodies from Thermo Fisher Scientific including goat anti-rat 488, goat anti-mouse 568, and goat anti-rabbit TRITC (T2769). The sections were mounted with Vectashield Mounting Medium H-1000 (Vector Laboratories, Burlingame, CA, USA). Five to ten brain sections from individual mice were assessed for each antibody. For BrdU staining, tissue sections were treated with 2 mol/L HCl for 20 min at 30 °C. The sections were then washed with PBS followed by incubation for 10 min at room temperature with 0.1 mol/L borate buffer (pH = 8.5). The sections were then washed with PBS and processed for immunofluorescent staining as above.

### Western blot analysis

To assess the protein expression of Phospho-Stat3 in the focal ischemic hemisphere, Western blot analysis was performed as described previously [35]. In brief, the frozen brain samples were homogenized in RIPA buffer containing protease inhibitor and 1 mM PMSF using a manual grinder. The homogenates were lysed in ice for 1 h and centrifuged at 12,000 rpm for 30 min at 4 °C. The supernatants were collected and the protein concentrations were measured using the BCA method with a Bio-Rad Protein Assay (Bio-Rad, Hercules, CA, USA). Equal amounts of protein (30 μg protein per lane) were loaded and separated by electrophoresis on 12% sodium dodecyl sulfate–polyacrylamide gels with a pre-stained protein ladder marker. The proteins were transferred onto the polyvinylidenedifluoride membranes (Millipore, Bedford, MA, USA). The membranes were blocked with TBST + 5% no-fat milk at room temperature (RT) for 1 h and then incubated overnight at 4 °C with rabbit anti-Phospho-Stat3 primary antibody. After washing with TBST, the membranes were incubated with secondary antibodies conjugated with horseradish peroxidase at RT for 2 h. The membranes were then washed and visualized by the Supersignal West Pico Trial kit (Pierce, Rockford, IL, USA). Blots were exposed to X-ray film (Fuji film, Tokyo, Japan). Anti-GAPDH antibody was used for normalization. The intensity of band was quantified, and relative levels of proteins in different lanes were compared using the NIH ImageJ program. All studies were performed three times with cerebral samples from three mice in each subgroup.

### Statistical analysis

All data were expressed as mean ± S.E.M and the differences between groups were evaluated by unpaired Student’s *t* test. All other measurements were analyzed statistically by one-way ANOVA followed by Bonferroni post test. Statistical significance was set at *p* < 0.05.

## Results

### GET-1 mice displayed more severe neurological deficits and larger infarct size after middle cerebral artery occlusion-induced cerebral ischemia

**Fig. 1:**
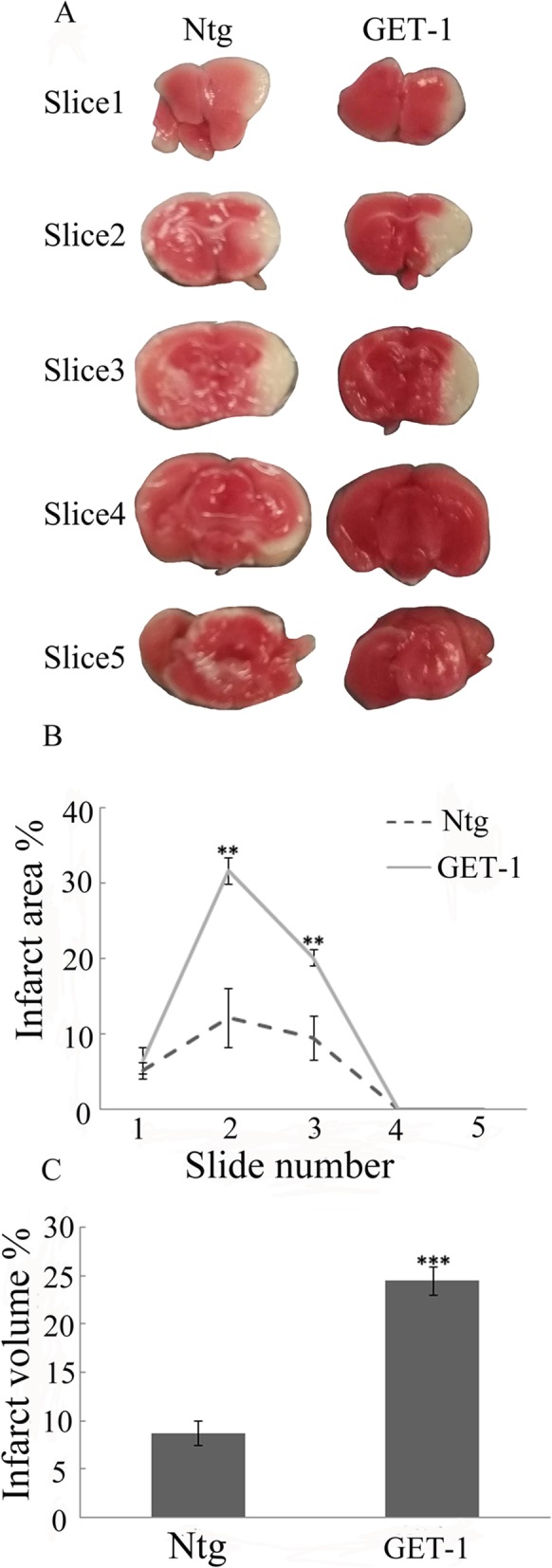
Infarct size in GET-1 and Ntg brains at 7 days after tMCAO. **a** Representative TTC-stained brain slices from Ntg and GET-1 mice after tMCAO. Note the larger white/pale regions indicating larger infarct areas in GET-1 mice (**b**) depiction of average infarct area as a percentage of the contralateral side in GET-1 and Ntg mice brain slices, **c** infarct volume % comparison between GET-1 and Ntg brains 7 days post tMCAO (***p < 0.001, **p < 0.01, ANOVA followed by Bonferroni’s test)

All the mice (Ntg and GET-1) subjected to 1 h of occlusion followed by either 1 or 7 days of reperfusion survived. The neurological assessment scores showed that there were more severe neurologic deficits in GET-1 mice than those in Ntg mice at 1 day (Table 1, *p* < 0.05) and 7 days after tMCAO (Table 2, *p* < 0.05). The neurological deficits of GET-1 mice did not show any significant improvement at 7 days compared to the results at 1 day after tMCAO. However, the neurological scores of all Ntg and GET-1 mice were restored to 0 score at 28 days after tMCAO. In addition, representative TTC-staining slices in Fig. 1a, b showed that infarct area (%) of slices 2 and 3 were significantly larger in GET-1 mice than observed in the Ntg mice at 7 days after stroke (*p* < 0.01 for slice #2; *p* < 0.01 for slice #3, Fig. 1a, b). Similarly, the mean infarct volume (% of normal side) in the GET-1 brains was also significantly larger than that of the Ntg mice brains (Fig. 1c).

**Table 1:** More severe neurologic deficits in GET-1 mice at 1 day after transient MCAO

Group	Neurologic scores				Average scores
	0	1	2	3	
Ntg (*n* = 11)	7	3	1	0	0.454
GET-1 (*n* = 11)	1	8	2	0	1.181^*^

The asterisk denotes significant difference between the mean neurological scores of Ntg and GET-1 mice, *p* < 0.01

**Table 2:** More severe neurologic deficits in GET-1 mice at 7 days after transient MCAO

Group	Neurologic scores				Average scores
	0	1	2	3	
Ntg (*n* = 11)	7	3	1	0	0.454
GET-1 (*n* = 11)	1	8	2	0	1.181^*^

The asterisk denotes significant difference between the mean neurological scores of Ntg and GET-1 mice, *p* < 0.01

### GET-1 mice showed increased SVZ cell proliferation in the ischemic brain

**Fig. 2:**
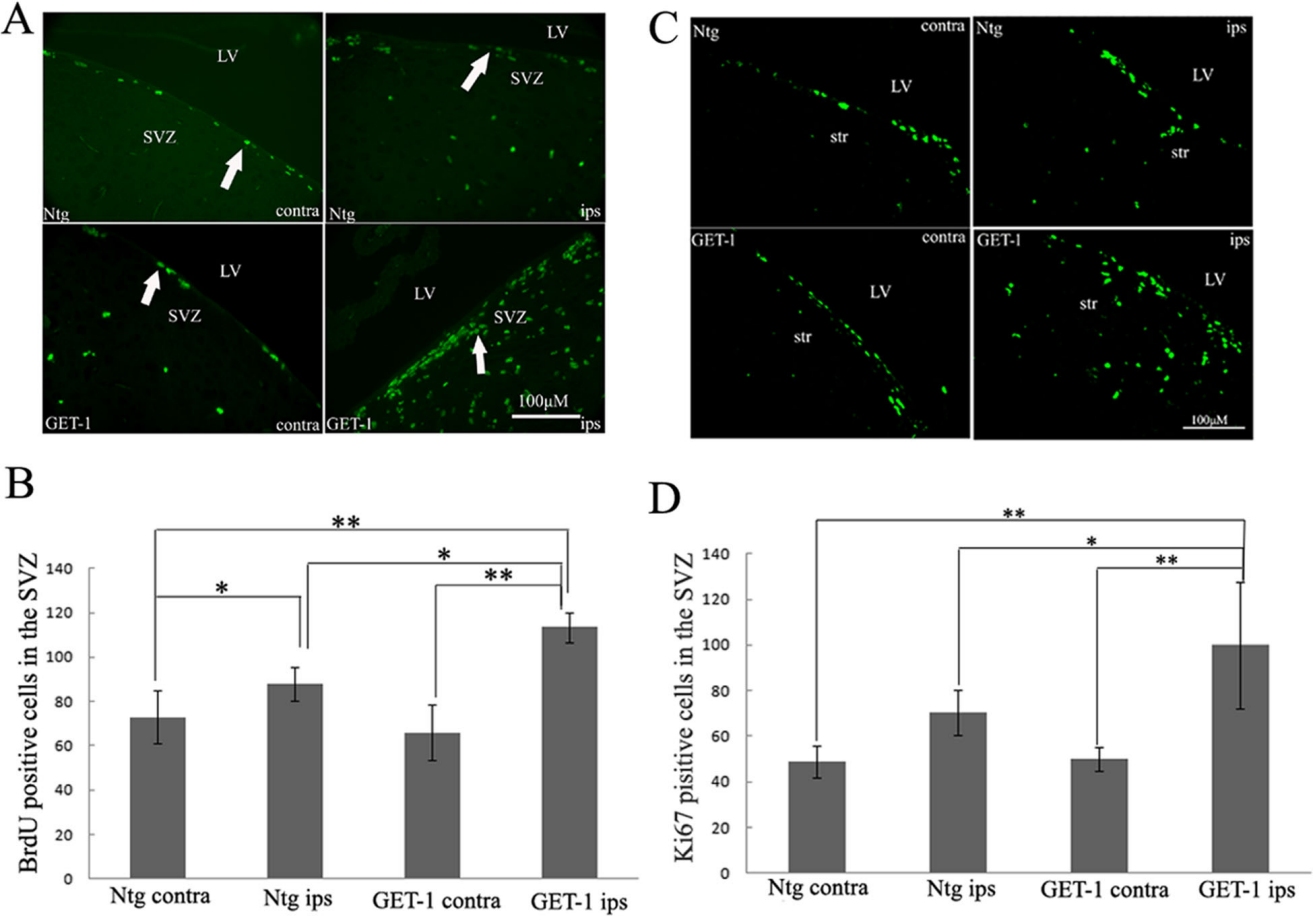
ET-1 overexpression promoted cell proliferation in the SVZ of mice following tMCAO photomicrographs of immunofluorescence staining showing **a** BrdU+ and Ki67+ cells **c** in the SVZ of Ntg and GET-1 mice, scale bar = 100 μm. **b** BrdU+ and **d** Ki67+ cell number was quantified in the contralateral and ipsilateral side of Ntg and GET-1 mice. Data is presented as mean ± S.E.M and was analyzed using the one-way ANOVA followed by Turkey post hoc comparisons. *p < 0.05, **p < 0.01. LV = lateral ventricle; SVZ = subventricular zone; str = striatum; ips = ipsilateral side of the ischemic brain. Scale bar = 100 μm

BrdU, which is incorporated in the S-phase cells in the cell cycle, is commonly used to detect cell proliferation. Ki-67, a nuclear protein expressed in all phases of the cell cycle except the resting phase, is used as a marker of proliferation in the initial phase of adult neurogenesis. To investigate whether overexpression of ET-1 in GET-1 brain affects SVZ cell proliferation after tMCAO, BrdU, and Ki-67 immunostaining were performed. The results shows that the numbers of BrdU (Fig. 2a, b, Additional file 2: Figure S2) and Ki-67 (Fig. 2c, d) positive cells increased significantly in the ipsilateral SVZ brains in GET mice when compared with those in Ntg mice at 7 days after tMCAO. In addition, there were more BrdU- and Ki67-positive cells in the ipsilateral side of the SVZ brains than those in the corresponding contralateral side of SVZ in both GET-1 and Ntg mice (*p < 0.05, **p < 0.01). Further analysis showed that the magnitude of the ipsilateral versus contralateral difference was greater in the GET-1 group than the Ntg group.

### ET-1 overexpression promoted NSC proliferation in SVZ after tMCAO

**Fig. 3:**
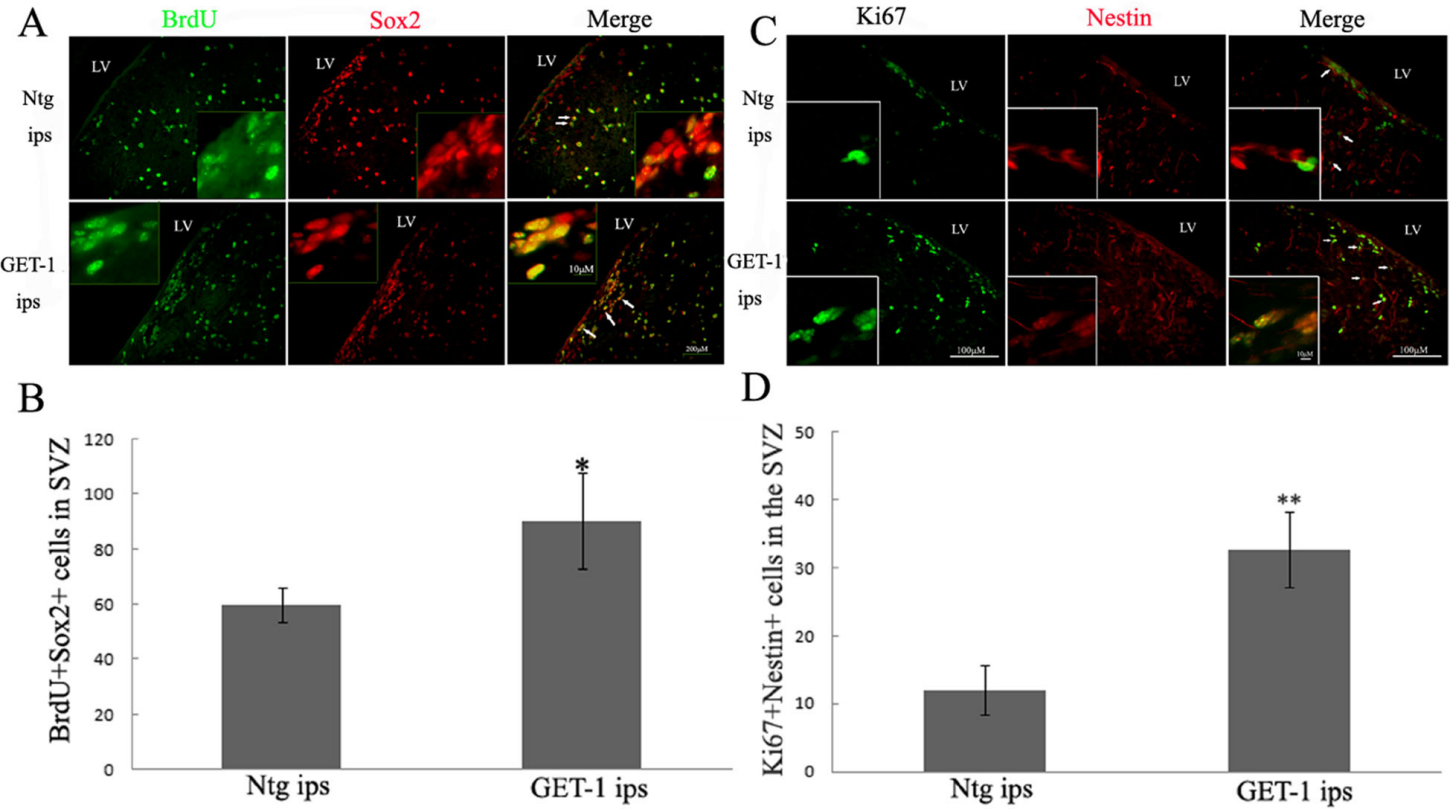
ET-1 overexpression promoted cell proliferation in the SVZ of mice following tMCAO. Double-labeled immunofluorescence staining showing the expression of **a** BrdU/Sox2; **c** Ki67/Nestin in SVZ of mice after tMCAO. The arrows indicate the positive signals inside cells. **b**, **d** The number of BrdU+Sox+ and Ki67+Nestin+ cells in the SVZ, respectively *p < 0.05 and **p < 0.01. LV = lateral ventricle; SVZ = subventricular zone; ips = ipsilateral side of ischemic brain

To assess whether these proliferating cells were indeed NSCs, BrdU and Ki67 double labeling with known phenotypic markers of stem cells, Sox2 or Nestin were performed. A representative slice in Fig. 3a, b is showing that in ipsilateral GET-1 mice brains, there were significantly more BrdU+Sox2+ positive cells compared to that in the corresponding side SVZ of Ntg mice at 7 days after tMCAO (Fig. 3a, b; *p < 0.05). A similar trend which is shown in Fig. 2 was also observed in the contralateral SVZ in which GET-1 and Ntg slices, there were also few BrdU+Sox2+ positive cell expression and there is no significant difference in the contralateral side of the brain between GET-1 and Ntg mice (figure was not shown), so there were more BrdU+Sox2+ double-labeled cells in the ipsilateral brain slices compared to that in the contralateral side brain of both GET-1 and Ntg mice which indicated that NSC proliferation was induced under ischemic stroke conditions. We view the significant increase of BrdU+Sox2+ positive cells in the ipsilateral side SVZ in GET-1 mice when compared with those of Ntg mice at 7 days after stroke suggested that ET-1 overexpression also promoted ischemia-induced NSC proliferation (Fig. 3a, b; **p < 0.01).

On the other hand, double staining of Ki67 and Nestin in Fig. 3c, d showed that there were almost three times more Ki67+Nestin+ positive cells in the ipsilateral slices at 7 days after tMCAO in GET-1 mice compared to Ntg (Fig. 3c, d; **p < 0.01). In addition, we also observed that there were less Ki67+Nestin positive cells in the contralateral side SVZ in both GET-1 and Ntg mice compared to respective tMCAO injured side (data not shown). Therefore, our results confirmed that ET-1 overexpression promotes ischemia-induced NSC proliferation.

### GET-1 mice did not affect SVZ cell migration in the ischemic brain after tMCAO

DCX is a microtubule-associated protein expressed by neuronal stem cells and immature neurons and is commonly used as a marker for migrating neuroblast and early differentiated neuron. In order to determine the effect of ET-1 overexpression on stem cell migration in the SVZ of ipsilateral hemisphere post tMCAO, BrdU/DCX double-immunostaining was performed. The results showed that there was no significant difference in the numbers of SVZ BrdU+DCX+ positive cells between the GET-1 and Ntg groups at 7 days after tMCAO (see Additional file 3: Figure S3, *p* > 0.05). This result appears to suggest that ET-1 overexpression did not affect SVZ cell migration in the ischemic brain after tMCAO.

### ET-1 overexpression stimulated astrogenesis in ischemic brain

**Fig. 4:**
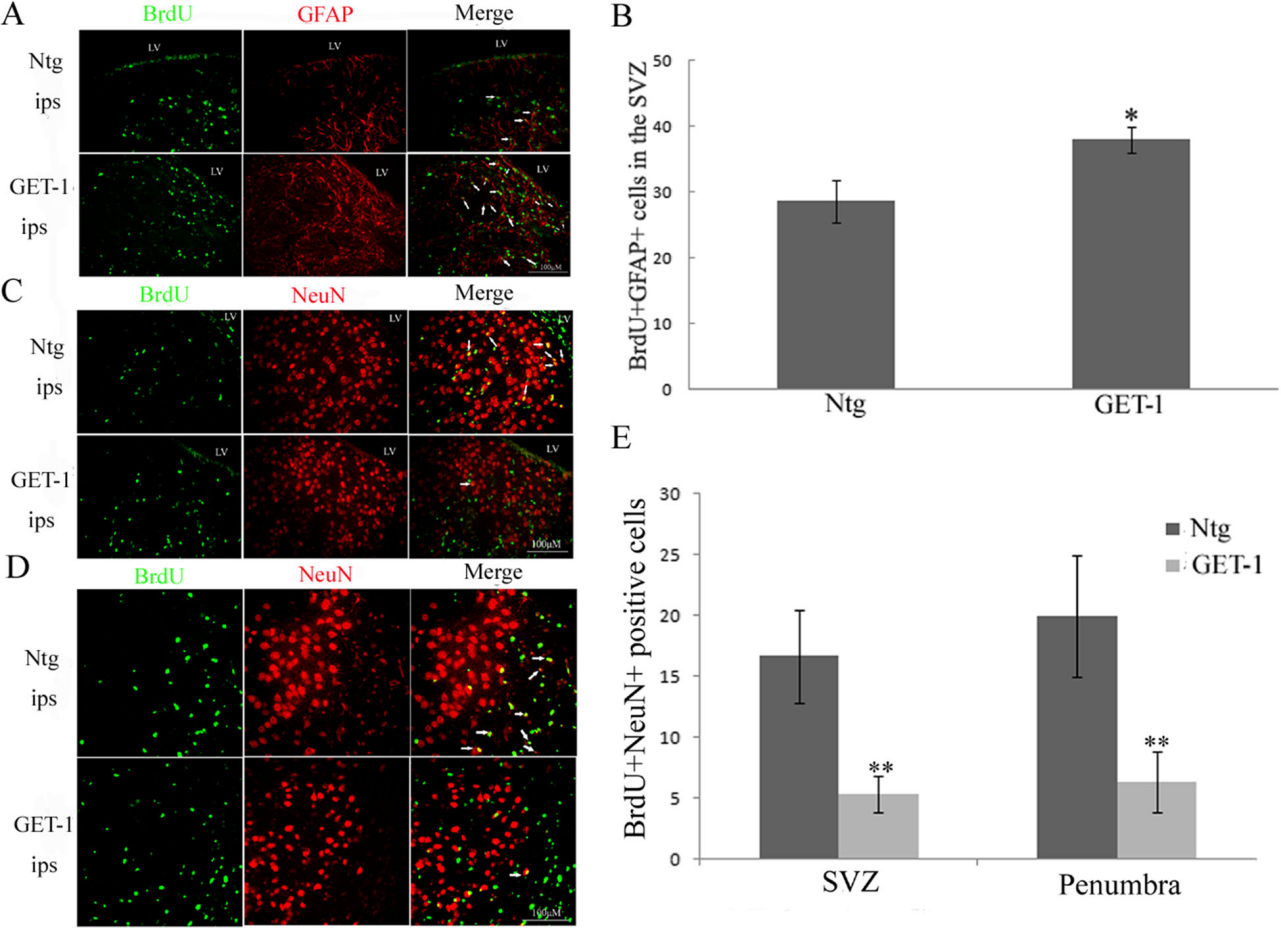
ET-1 overexpression influences astrogenesis post tMCAO. *ET-1 overexpression stimulates astrogenesis in the the SVZ of the ischemic* brain at 28 days and activates phosphorylation Stat3 expression at 7 days in the ischemia brain following tMCAO. **a** Images show immunostaining for BrdU+GFAP+ cells in SVZ of Ntg and GET-1 mice 28 days post tMCAO. **b** bar graph depicting the high number of BrdU/GFAP co-labeled cells in ipsilateral SVZ of GET-1 than Ntg mice; **c** Images show double immunostaining for BrdU and NeuN iin SVZ cells of Ntg and GET-1 mice 28 after tMCAO. **d** Images show double immunostaining for BrdU and NeuN iin penumbra region cells of eNtg and GET-1 mice 28 days post tMCAO. **e** Bar graphs depicting the number of BrdU/NeuN co-labeled cells in ipsilateral SVZ and penumbra region of GET-1 mice than Ntg. Scale bar = 100 μm

In order to investigate whether these proliferating NSCs could differentiate into mature neurons or astrocytes, and also, if they will show beneficial or detrimental effects to the ischemia injured brain, BrdU double labeling of the cells with either NeuN or GFAP was performed. Interestingly, at 28 days after tMCAO, double immunofluorescent staining for BrdU and GFAP showed that most of BrdU-stained cells expressed GFAP in the ipsilateral side SVZ of GET-1 mice (Fig. 4a, b, white arrows). On the contrary, very few such double stained cells were observed in the Ntg wild type control ipsilateral SVZ (Fig. 4a, b, white arrows).

Furthermore, only a few BrdU-positive cells co-labeled with NeuN in GET-1 mice could be observed (Fig. 4c, e white arrows). However, we observed that there were more BrdU+ cells that co-localized with NeuN in the ipsilateral side of Ntg mice SVZ while less co-labeled with GFAP (Fig. 4a, c, white arrows). In agreement with the SVZ data, Fig. 4d, e shows that there were more BrdU-positive cells that co-localized with NeuN in the penumbra region in the ipsilateral side of Ntg mice than those in GET-1 mice (Fig. 4d, e, white arrows). The above results indicate that ET-1 overexpression-induced astrogenesis in SVZ following tMCAO.

### Astrocytic ET-1 overexpression activates phosphorylation of Stat3 (p-Stat3) in the ischemic brain

**Fig. 5:**
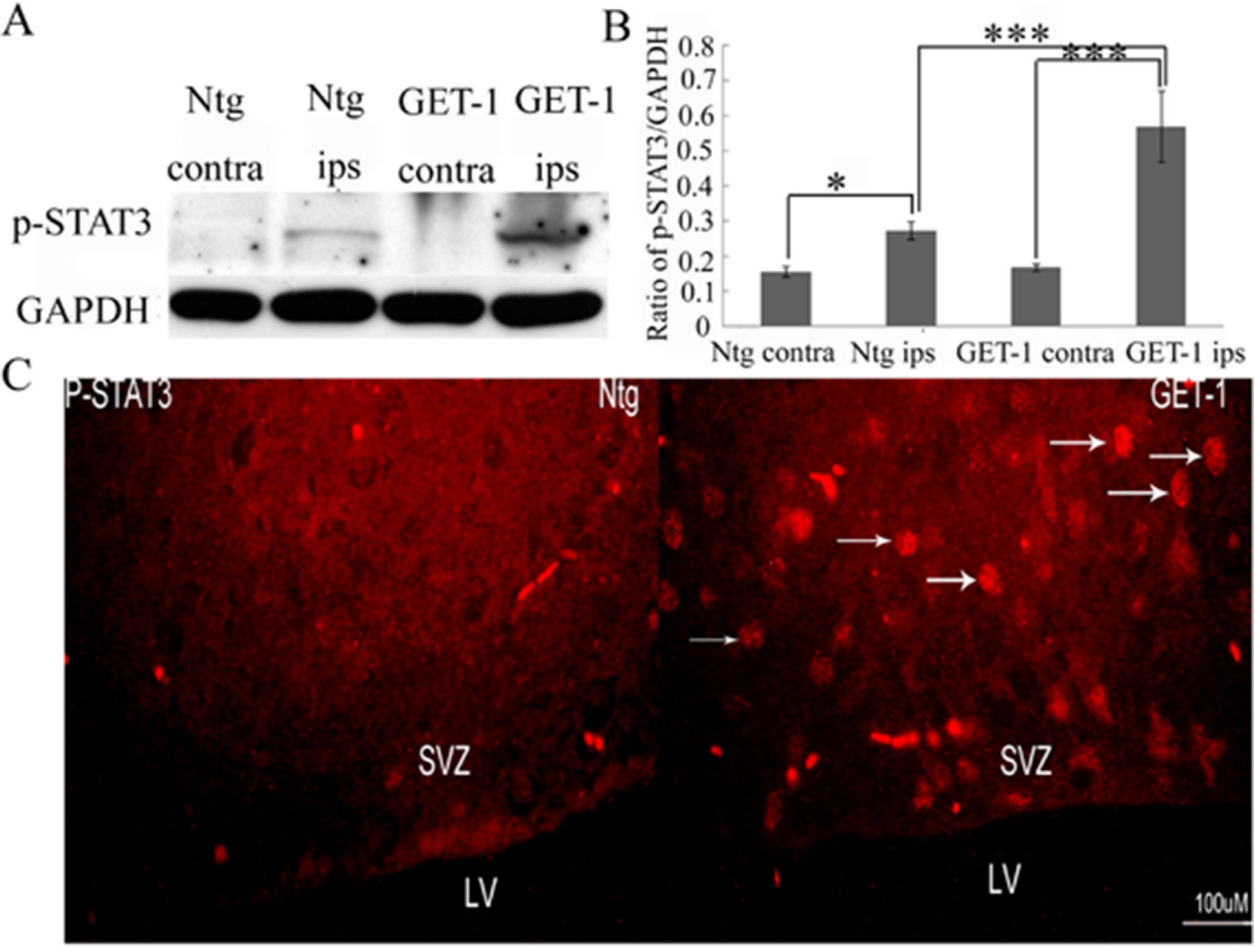
Stat 3 phosphorylation after tMCAO. **a** Representative immunoblot results at 7 days after tMCAO. **b** Semi-quantitative analysis of p-Stat3 protein demonstrating that p-Stat3 expression was upregulated after tMCAO both in Ntg and GET-1 mice. p-Stat3 expression was robustly upregulated in the ipsilateral in GET-1 mice brain tissue than in Ntg mice 7 days after tMCAO. *N* = 3 per group pooled together and assayed thrice. *p < 0.05, ***p < 0.001; Contra = contralateral side of the ischemic brain; Ips = ipslateral side of the ischemic brain. **c** Representative images of p-Stat3 expression in ipsilateral SVZ of each group (red, indicated by white arrows). Scale bar = 100 μm; LV = lateral ventricle, SVZ = subventricular zone

Previous studies showed that activation of the NSCs Jak-Stat3 pathway promotes astrogenesis [36–39]. Jak2 and Stat3 activation has previously been shown to contribute to neuronal damage following transient focal cerebral ischemia [40]. Therefore, we speculated that astrocytic ET-1 overexpression might stimulate astrogenesis through Stat3 signaling pathway after tMCAO. Firstly, p-Stat3 expression in Ntg and GET-1 mice 7 days after tMCAO was investigated by Western-blot and immunofluorescence staining. Western-blot analysis showed that p-Stat3 expression was increased in both Ntg and GET-1 mice at 7 days after tMCAO (their corresponding contralateral vs ipsilateral sides are shown in Fig. 5a, b). We also noted that p-Stat3 expression was significantly upregulated in the ipsilateral side in the GET-1 brain than that in the Ntg brain (Fig. 5c). Immunofluorescence staining demonstrated that p-Stat3 expression localized in the cell nucleus. Consistent with the above Western-blot results, p-Stat3 staining was more intense in the ipsilateral side in GET-1 mice than that in Ntg mice at 7 days after stroke (Fig. 5c). The results stresses that overexpression of ET-1 significantly induces an increase of Jak2/Stat3 activation, phosphorylation of Stat3 and therefore marked astrogenesis in GET-1 mice.

### Neuroprotective effects of AG490 on neurologic deficits and infarct size after tMCAO

**Fig. 6:**
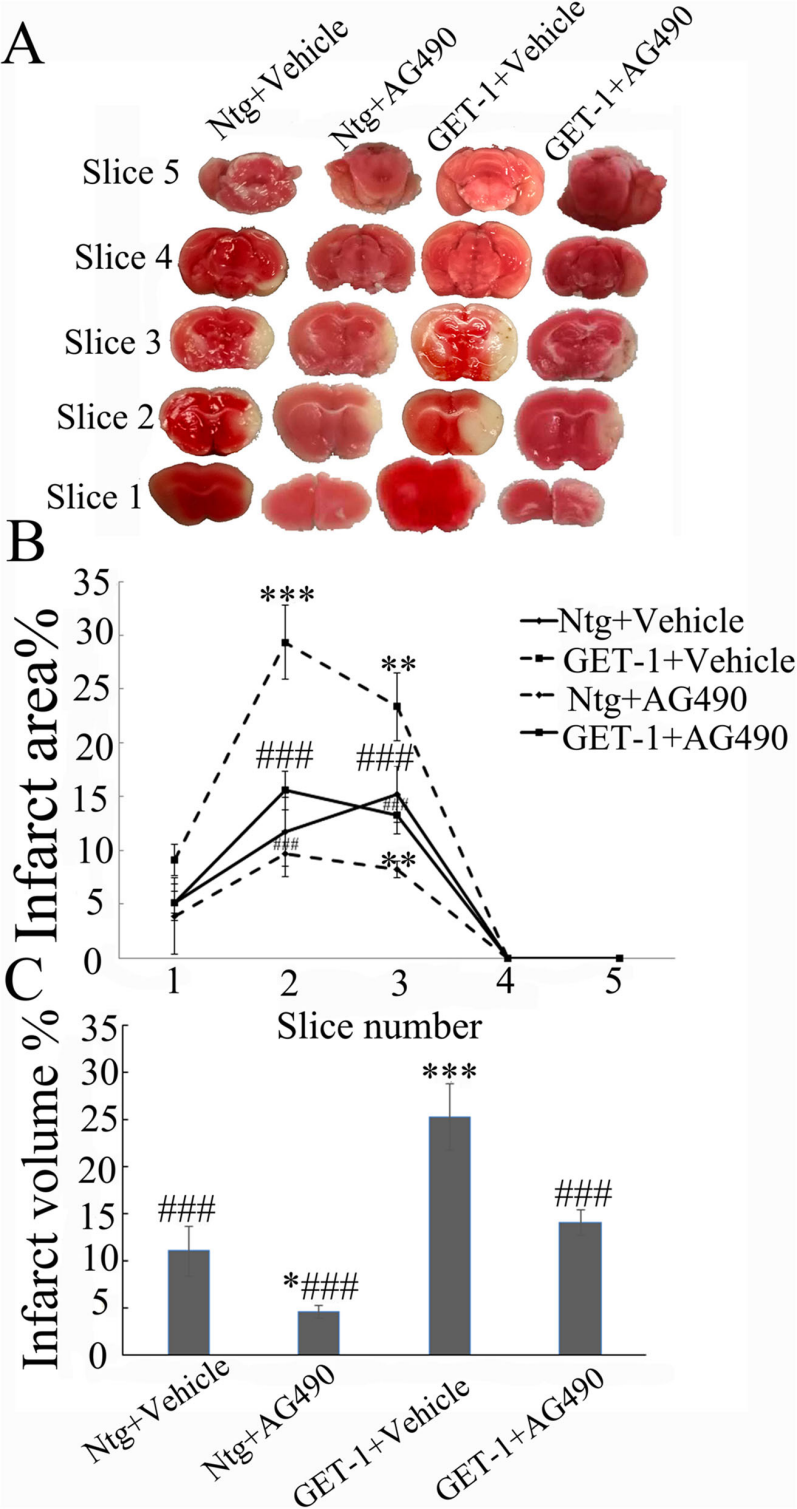
Brain infarct size at 7 days in the Ntg and GET-1 brains after AG490 treatment. **a** Representative TTC-stained brain slices from Ntg and GET-1 mice after tMCAO. Note the larger white regions indicating larger infarct areas **b** infarct area expressed as a percentage of the contralateral side in each group (**p < 0.01, ***p < 0.001 V. Ntg + vehicle group; ###*p* < 0.001 V. GET-1 + vehicle group). **c** Infarct volume expressed as a percentage of the contralateral side in each group (*p < 0.05, ***p < 0.001 V. Ntg + vehicle group; #*p* < 0.05, ###*p* < 0.001 V. GET-1 + saline)

In order to determine whether pretreatment with a specific inhibitor of JAK2/STAT3 (AG490) would ameliorate the brain damage in ischemic stroke, we administered AG490 prior to tMCAO in Ntg and GET-1 mice. All the mice of both genotypes survived the whole course of the experiments. As shown in Fig. 6a, b, TTC-staining analyses showed that the infarct areas (%) of brain slices #2 and #3 in the vehicle-treated GET-1 mice were significantly increased when compared with those in the vehicle-treated Ntg mice at 7 days after stroke. After AG490 treatment, the infarct areas of brain slice 3 in Ntg mice were significantly reduced, while that of brain slices 2 and 3 in GET-1 mice were also significantly reduced (Fig. 6a, b). Similarly, the mean infarct volume (%) in the vehicle-treated GET-1 mice were significantly increased when compared with those in the vehicle-treated Ntg mice at 7 days after stroke. After AG490 treatment, the infarct volume in both Ntg and GET-1 mice were significantly reduced (Fig. 6c).

Neurological function score assessment showed that there were more severe neurological deficits in GET-1 + vehicle group than that in Ntg + vehicle group at 1 day (Table 3) and 7 day (Table 4) after tMCAO (***p < 0.001 V. Ntg+ vehicle group). The neurological deficits of GET-1 + AG490 group were significantly improved compared to that in GET-1 + vehicle group at 1 day and 7 days after tMCAO (#*p* < 0.05 V. GET-1 + vehicle group). The neurologic deficits that the Ntg and GET-1 mice suffered as a result of 1 h tMCAO had largely disappeared at day 28.

**Table 3:** AG490 treatment reduces neurologic deficits in GET-1 and Ntg mice at 1 day after tMCAO

Group	Neurological scores				Average scores
	0	1	2	3	
Ntg + vehicle (*n* = 15)	9	6	0	0	0.4
Ntg + AG490 (*n* = 8)	7	1	0	0	0.125
GET-1 + vehicle (*n* = 17)	1	9	7	0	1.35^***^
GET-1 + AG490 (*n* = 9)	4	3	2	0	0.78^#^

The asterisk denotes significant difference between the mean neurological scores of Ntg and GET-1 mice, *p* < 0.01

**Table 4:** AG490 treatment reduces neurologic deficits in GET-1 and Ntg mice at 7 days after tMCAO

Group	Neurological scores				Average scores
	0	1	2	3	
Ntg + vehicle(*n* = 15)	9	6	0	0	0.4
Ntg + AG490 (*n* = 8)	7	1	0	0	0.125
GET-1 + vehicle (*n* = 17)	1	9	7	0	1.35^***^
GET-1 + AG490 (*n* = 9)	4	3	2	0	0.78^#^

Numbers of mice having various degrees of neurologic deficits after tMCAO. Numbers 0–3 in the top row represent severity of neurologic deficits with 0 being normal and 3 being the most severe

*Ntg* nontransgenic, *MCAO* middle cerebral artery occlusion

The asterisk denotes significant difference between the mean neurological scores of Ntg and GET-1 mice, *p* < 0.01

### AG490 treatment inhibited astrogenesis at 28 days in SVZ ipsilateral side of GET-1 and Ntg mice after tMCAO

**Fig. 7:**
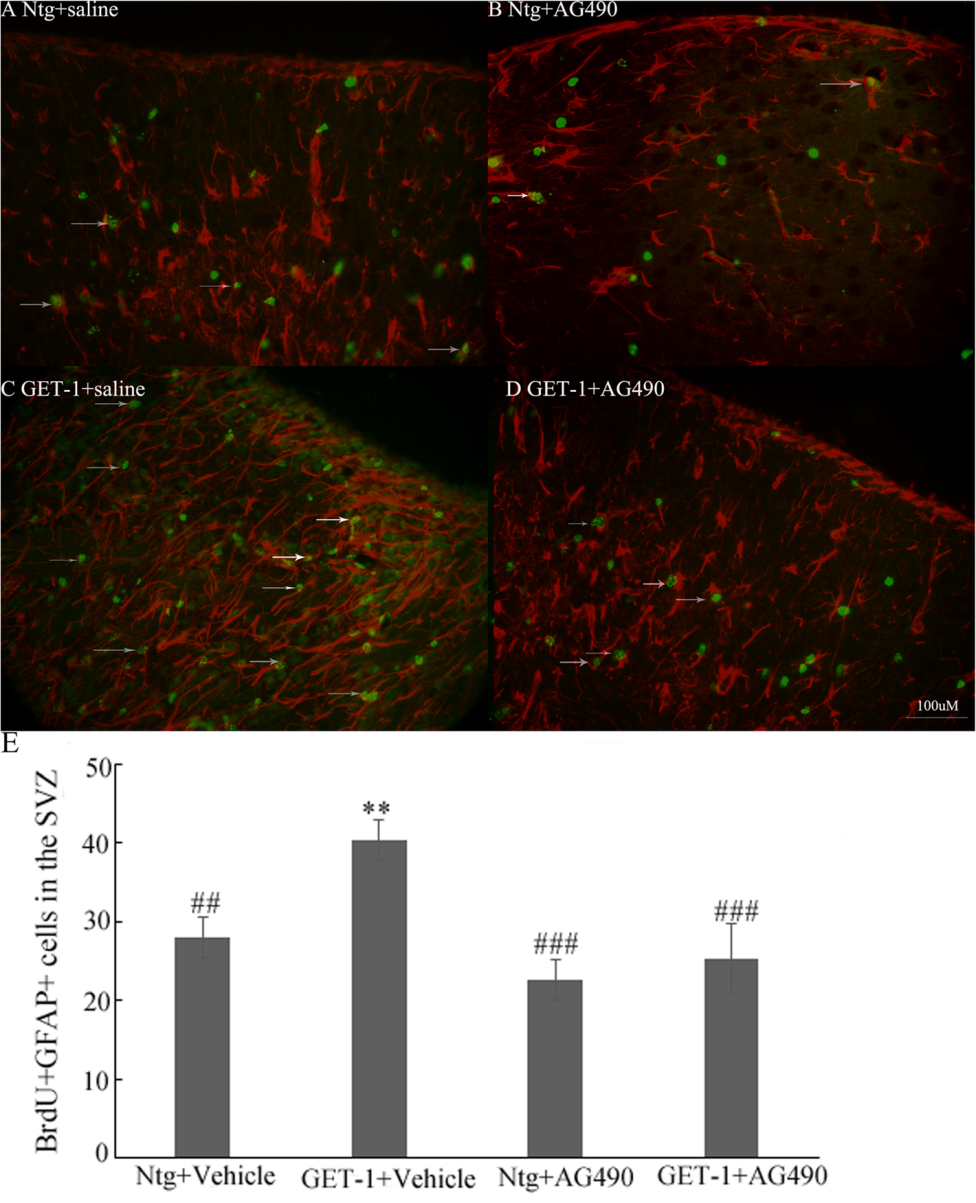
AG490 treatment inhibited astrogenesis at 28 days in SVZ ipsilateral side of GET-1 and Ntg mice after tMCAO. Images show representative immunostaining profile for BrdU+GFAP+ cells in ipsilateral SVZ of Ntg (**a**-control Vs **b** AG490 treated) and GET-1 (**c**-control Vs **d** AG490 treated) mice 28 days after tMCAO. (positive signals indicated by white arrows, red = GFAP, green = BrdU). Scale bar = 100 μm. **e** Bar graphs depicting the number of BrdU/GFAP co-labeled cells in ipsilateral SVZ of Ntg and GET-1 mice after AG490 and vehicle treatment 28 days after tMCAO. ** *p* < 0.05 V.Ntg+Vehicle group, ## *p* < 0.01 ### *p* < 0.001V.GET-1+Vehicle group

To further verify that the observed reduced brain damage in GET-1 brain after AG490 treatment was possibly due to alternation of astrogenesis in ischemic stroke, double labeling for BrdU, and GFAP in Ntg and GET-1 mice after AG490 treatment was performed. BrdU and GFAP co-staining showed that there were more BrdU-co-localized with GFAP cells in the ipsilateral side of SVZ at 28 days after tMCAO in GET-1 + vehicle mice that that in Ntg + vehicle mice. After AG490 treatment, BrdU-positive cells which also expressed GFAP in the ipsilateral SVZ were significantly reduced in both GET-1 and Ntg mice (Fig. 7). There were no observable differences in the number of BrdU+NeuN co-localized cells in the ipsilateral SVZ between Ntg and GET-1 mice after AG490 treatment (data not shown). Taken together, these findings indicated that astrocytic endothelin-1 overexpression promoted neural progenitor cell proliferation and differentiation into astrocytes via the Jak2/Stat3 pathway in murine models of stroke.

## Discussion

In our previous study, we have demonstrated that mice overexpressing ET-1 (GET-1) exhibited more severe neurological deficits coupled with water homeostasis dysregulation, cerebral edema, and BBB integrity impairment, all of which contributed to more severe ischemic brain injury [9]. We also demonstrated that astrocytic ET-1 played a deleterious role in cognitive function and progressive neurodegeneration associated with a short-term ischemia/longer-term reperfusion [17]. In the present study, we further revealed the role of astrocytic ET-1 under mild ischemic condition (1-h cerebral occlusion) and long-term reperfusion injury (7 days and 28 days). In this study, we also elucidated the mechanism and signaling pathway involved in astrocytic endothelin-1-mediated brain damage. Our present data showed that GET-1 mice subjected to 1-h mild ischemic injury followed by long-term reperfusion exhibited more severe neurological deficits when compared to those of Ntg mice within the first week after injury. Our data further demonstrated that astrocytic ET-1 promoted proliferation of neural progenitor cells and their differentiation into astrocytes after stroke via the Jak2/Stat3 pathway and thus contributed to more severe brain damage after stroke.

To date, systemic thrombolysis with rt-PA has so far proved to be the only effective way for stroke treatment in the clinic [41]. Despite numerous studies focusing on protecting ischemic neurons and preventing neuronal loss, various clinical trials have failed to demonstrate many targets including antioxidants, calcium channel blockers, glutamate receptor blockers, and neurotrophic factors [42]. In the early times, the focus has been on neuronal mechanisms of injury and benefits in treating stroke. However, recent attention is now on developing alternative stroke treatments which focus on non-neuronal cell types such as astrocytes dysfunction [43, 44]. Astrocytes are the most abundant cell type in the mammalian nervous system and they play key roles in maintaining normal CNS physiological and pathological processes [22, 31, 33]. Astrocytes play an important but ambivalent role in the pathogenesis of stroke [45–49]. Stroke causes an initial process of local scar formation that limits damage, but it also limits tissue repair by impeding new connections and angiogenesis [50]. Astrocytes also regulate synapses and have links to blood vessels with their cell processes where they release neuro-immune mediators in response to damage [50]. Astrocytes are the main component of the glial scar and many studies have shown that decreased astrogliosis would reduce infarct size [43].

A selective cyclin kinase inhibitor suppresses astroglial proliferation and glial scar formation as well as decreases neuronal cell death in the ischemic boundary zone and hippocampal CA1 region after tMCAO [51]. In addition, treatment with alpha-melanocyte-stimulating hormone [52] and caffeic acid [53] reduce infarct size and astrogliosis. Knockdown of Ski decreased the reactive astrocyte proliferation in vitro induced by oxygen-glucose deprivation/reoxygenation [54]. Thus, results from these studies suggest that stroke treatments are often accompanied by attenuating astrocyte proliferation or astrogliosis. In agreement with the previous studies, the present study also showed that astrocytic endothelin-1 overexpression promotes astrogenesis which contributes to more severe ischemic brain injury in murine models of stroke during the first week. The severity of ischemic brain injury noted at days 1 and 7 post tMCAO could be explained by the vasocontractile effects of ET-1 which likely impairs collateral circulation during the evolution of stroke. On the other hand, inhibiting astrogenesis with AG490 treatment reduced in infarct size and severity of neurological deficits after tMCAO. These observations suggest that reduced ET-1 activity from astrocytes is neuroprotective and could be pursued further in future studies.

The neurogenesis in the adult mammalian brain has been described as occurring and preserved in two specific zones, the neural niches; the subventricular zone (SVZ) in the lateral wall of the lateral ventricle and the subgranular layer of the dentate gyrus of the hippocampus [55]. It has recently been found that lesioned astrocytes induce adult neural stem/progenitor cell differentiation into astrocytes in vitro [56–60]. Furthermore, co-culturing NSCs with lesioned astrocytes reveals that several of these NSC-derived astrocytes participate in glial scar formation in vitro [61]. These findings demonstrate that astrocytes play key roles in the stimulation of neural niches after cerebral ischemia and possibly in the regulation of adult neurogenesis under physiological conditions. However, the substances secreted by astrocytes after brain injuries such as stroke (which can regulate neural stem cell proliferation and differentiation) are complex and remain poorly understood.

In an oxygen-glucose deprivation/reperfusion (OGD/R) model, a damage-associated molecular-patterning molecule called high-mobility group box 1 (HMGB1) is released by astrocytes [62]. HMGB1 is critical for NSC/neuro-progenitor proliferation during brain development. It was demonstrated that HMGB1 released by OGD/R astrocytes promotes NSC proliferation through activation of the PI3K/protein kinase B (PI3K/Akt) signaling pathway [63]. Under ischemic stroke conditions, endothelin-1 (ET-1) is upregulated in astrocytes [16]. Our previous and present data further confirmed that under mild ischemic injury with long-term reperfusion, mice overexpressing ET-1 (GET-1 mice) exhibited severer neurological deficits when compared to that of wild type Ntg mice. Our previous in vitro study also showed that ET-1 released by the astrocytes induces proliferation and differentiation of astrocytes through the Erk and JAK/STAT pathways under H/I stress conditions [64]. To our knowledge, the present study is the first to use a transgenic mice overexpressing astrocytic ET-1 to investigate the role of astrocytic ET-1 on neural stem cell fate under mild ischemic stroke condition. We have demonstrated that lesioned astrocytes induced de novo astrocytic differentiation of adult neural stem/progenitor cells via ET-1 secretion in a transgenic mouse model of stroke. We have also showed that astrocytic ET-1 induced Erk and JNK pathways as well as PI3K/Akt pathway in our GET-1 and Ntg mice after tMCAO, although the difference was not statistically significant (data not shown). These observations suggested that the Erk/JNK and PI3K/Akt pathways may be ET-1 independent under mild stroke conditions.

Numerous studies have also reported the signaling pathways involved in the induction of astrocyte specification/differentiation under pathological conditions. Astrocyte differentiation is thought to be induced via cytokine and/or BMP signaling pathways. Cytokines (LIF or CNTF) induce dimerization of the LIF receptor (LIFR) with co-receptor gp130, leading to phosphorylation and activation of Janus kinases (Jaks) [36]. Receptor dimerization also creates docking sites for the transcription factor Stat3, which is tyrosine phosphorylated by Jaks. The phosphorylated Stats dimerize and move into the nucleus, recruiting p300 and binding to specific sequences in the GFAP promoter, which indicated that Jak2/Stat3 signaling pathways involved in the induction of astrocyte differentiation [38]. In previous studies, the Notch1–STAT3–ETBR signaling axis that controls reactive astrocyte proliferation after brain injury has also been reported [65]. We recently detected Notch1 expression in GET-1 and Ntg mice after stroke, and the results demonstrated that there is no difference of Notch1 expression in Ntg and GET-1 mice in both ipsilateral and contralateral sides at 7 days after tMCAO (data not shown). Jak 1 and Stat3 have been reported to play important roles during rodent brain development [66, 67].

Activation of the JAK-STAT3 pathway in NSCs to promote astrogenesis has been studied before [36, 38, 39]. However, under cerebral ischemic conditions, JAK2 and STAT3 activation contribute to neuronal damage [40]. The neuroprotective effects of the JAK-STAT signaling pathway inhibitor have also been reported in the treatment of focal cerebral ischemia/reperfusion injury in rats [68]. Previously, it was demonstrated that Jak1 and Stat3 mediated pathway is activated in astrocytes following a transient focal cerebral ischemia (50-min occlusion). Results from this study suggest that Jak1 mediates Stat3 nuclear translocation in astrocytes where it, in turn, regulate gliogenesis [69]. In the present study, we demonstrated that the JAK/STAT pathway was activated in GET-1 mice after tMCAO. We further administered a specific inhibitor of JAK2/STAT3 (AG490) which resulted in inhibition of Jak2/Stat3-mediated astrocyte differentiation. Our findings are in agreement with another study which shows that SOCS1-JAK2-STAT3 pathway inhibition using AG490 reduces neuronal death after ischemic occlusion by attenuating oxidative stress and promoting injured-neuronal ultra-structure repair [70]. Our study findings further suggest that not only Jak1, but also Jak2 may be involved in gliogenesis and astrocyte differentiation from neural stem cells after ischemic stroke. Therefore, inhibition of Jak2/STAT3 could prevent neuronal cell death by reducing oxidative stress as well as astrogenesis-induced complications. It has been reported that pretreatment with a specific inhibitor of JAK2/STAT3, AG490, prevents ET-1-mediated resistin mRNA increase and also reduces ET-1-stimulated phosphorylation of STAT3 [71]. These studies have demonstrated that the JAK2/STAT3 pathway is involved in cerebral ischemia injury and astrocyte differentiation. In agreement, our present study found that the severe brain damage in astrocytic ET-1 overexpression after stroke is due to dysregulation of neurogenesis by promoting astrocyte differentiation. For the first time, we have shown that JAK2-STAT3 signaling pathway was involved in astrocyte differentiation after a stroke. Our experimental data also showed that endothelin B receptor co-localized with NSC marker Nestin (Additional file 4: Figure S4) in GET-1 mice and that was highly expressed in the ipsilateral side after tMCAO than that in contralateral side. Future studies should explore the occurrence of the role of the other endothelin receptor A, if any, in post stroke pathogenesis.

Overall, the findings of the present study further confirmed our hypothesis that astrocytic endothelin-1 overexpression promotes neural progenitor cell proliferation and differentiation into more astrocytes after stroke via the Jak2/Stat3 pathway. However, the exact mechanism through which astrocytic endothelin-1 affects the Jak2/Stat3 pathway in a murine stroke model need would benefit from further investigation in the future.

## Conclusions

The present study reports, for the first time, that astrocytic ET-1 overexpression in mice displayed more severe neurological deficits and larger infarct after stroke due to dysregulation of neurogenesis. The alteration of the stem cell niche, which promotes neural progenitor cell proliferation and differentiation into astrocytes and act via the Jak2/Stat3 signaling pathway involved dysregulation of neurogenesis. It is important to investigate further and understand the relationship between astrocytic ET-1 and its propensity to stimulate neurogenesis or astrogenesis understroke condition, so that potential targets could be identified and used to develop potential therapeutic drugs for stroke.

## Supplementary information

**Additional file 1:** **Figure S1.** Schematic representation of the experiment set up: tMCAO modeling, BrdU injections, AG490 administration, and functional assessments. (A) Flow chart of the BrdU labelling experiment. Mice underwent tMCAO at day 0, followed by BrdU was administrated by intraperitoneal injection for 5 days. Mice were sacrificed for immunostaining at 7d and 28d. (B) Flow chart of the BrdU labelling and AG490 treatment experiment. Mice underwent MCAO at day 0, followed by BrdU was administrated by intraperitoneal injection for 5 days. And AG490 was administrated by intraperitoneal injection for 5 times at 30min before surgery, 1st, 2nd, 4th, 6th day after surgery respectively, mice were sacrificed for immunostaining at 7d and 28d.

**Additional file 2:** **Figure S2.** IHC staining of BrdU. in the SVZ of Ntg and GET-1 mice after tMCAO. Photomicrographs of immunofluorescence staining showing BrdU+ in the contralateral and ipsilateral SVZ of Ntg and GET-1 mice. LV=lateral ventricle; SVZ=subventricular zone; str=striatum;Contra=contralateral side of the ischemic brain; ips=ipsilateral side of the ischemic brain. Scale bar=100 μm.

**Additional file 3:** **Figure S3.** Images show immunostaining for BrdU and DCX in SVZ cells of Ntg and GET-1 mice 28 days post tMCAO. GET-1 mice did not affect SVZ cell migration in the ischemic brain after tMCAO. Neuroblast migration from the SVZ through the CC to the peri-infarct cortex BrdU+DCX+(neuroblast) double-immunostaining and area quantification in the dorsolateral ventricle (DL) area at day 7 after tMCAO in the contralateral and ipslateral side of Ntg and GET-1 mice. Scale bar=100 μm; Str=striatum; SVZ=subventricular zone; Contra=contralateral side of ischemia brain; Ips= ipsilateral side of ischemia brain. CC= Corpus callosum.

**Additional file 4:** **Figure S4.** Images show immunostaining for ET-B+Nestin+ in SVZ cells of GET-1 mice 7 days post tMCAO. LV=lateral ventricle; SVZ=subventricular zone; Contra=contralateral side of the ishchemic brain; ips= ipsilateral side of the ischemic brain. Scale bar=100 μm.

**Additional file 5:** **Figure S5.** Representative read out of cerebral blood flow as monitored during tMCAO.

## Data Availability

The dataset used during the current study is stored in a secured research data server at Hong Kong University. The datasets used are available from the corresponding author upon reasonable request.

## Abbreviations

BrdU: Bromodeoxyuridine
CNS: Central nervous system
DCX: Doublecortin
ET-1: Endothelin-1
GET-1: Transgenic mice with astrocytic ET-1 overexpression
GFAP: Glial fibrillary acidic protein
NeuN: Neuron-specific nuclear protein
NPCs: Neural progenitor cells
NSC: Neural stem cell
Ntg: Non-transgenic
OGD/R: Oxygen glucose deprivation/reperfusion
p-Stat3: Phospho-Stat3
SVZ: Subventricular zone
tMCAO: Transient middle cerebral artery occlusion
TTC: 2,3,5-Triphenyltetra-zolium chloride
