# Supplementary figures and images for: Astrocytic endothelin-1 overexpression promotes neural progenitor cells proliferation and differentiation into astrocytes via the Jak2/Stat3 pathway after stroke
Source: J Neuroinflammation. 2019 Nov 16;16:227. doi: 10.1186/s12974-019-1597-y (PMC6858703; doi:10.1186/s12974-019-1597-y)

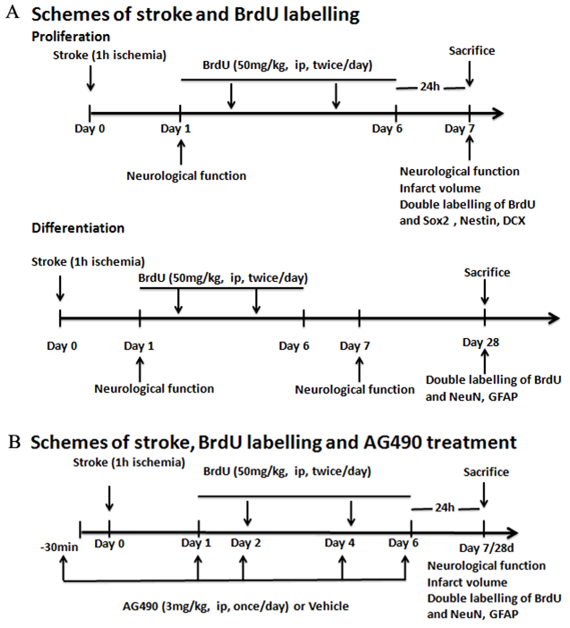

Supplement: Supplementary file 1 — Additional file 1: Figure S1. Schematic representation of the experiment set up: tMCAO modeling, BrdU injections, AG490 administration, and functional assessments. (A) Flow chart of the BrdU labelling experiment. Mice underwent tMCAO at day 0, followed by BrdU was administrated by intraperitoneal injection for 5 days. Mice were sacrificed for immunostaining at 7d and 28d. (B) Flow chart of the BrdU labelling and AG490 treatment experiment. Mice underwent MCAO at day 0, followed by BrdU was administrated by intraperitoneal injection for 5 days. And AG490 was administrated by intraperitoneal injection for 5 times at 30min before surgery, 1st, 2nd, 4th, 6th day after surgery respectively, mice were sacrificed for immunostaining at 7d and 28d. [file 12974_2019_1597_MOESM1_ESM.tif]

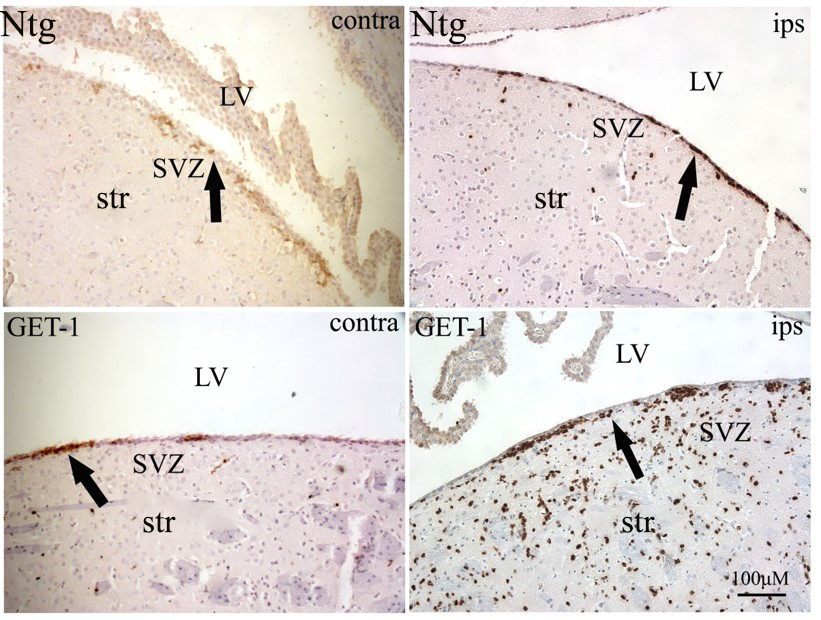

Supplement: Supplementary file 2 — Additional file 2: Figure S2. IHC staining of BrdU. in the SVZ of Ntg and GET-1 mice after tMCAO. Photomicrographs of immunofluorescence staining showing BrdU+ in the contralateral and ipsilateral SVZ of Ntg and GET-1 mice. LV=lateral ventricle; SVZ=subventricular zone; str=striatum;Contra=contralateral side of the ischemic brain; ips=ipsilateral side of the ischemic brain. Scale bar=100 μm. [file 12974_2019_1597_MOESM2_ESM.tif]

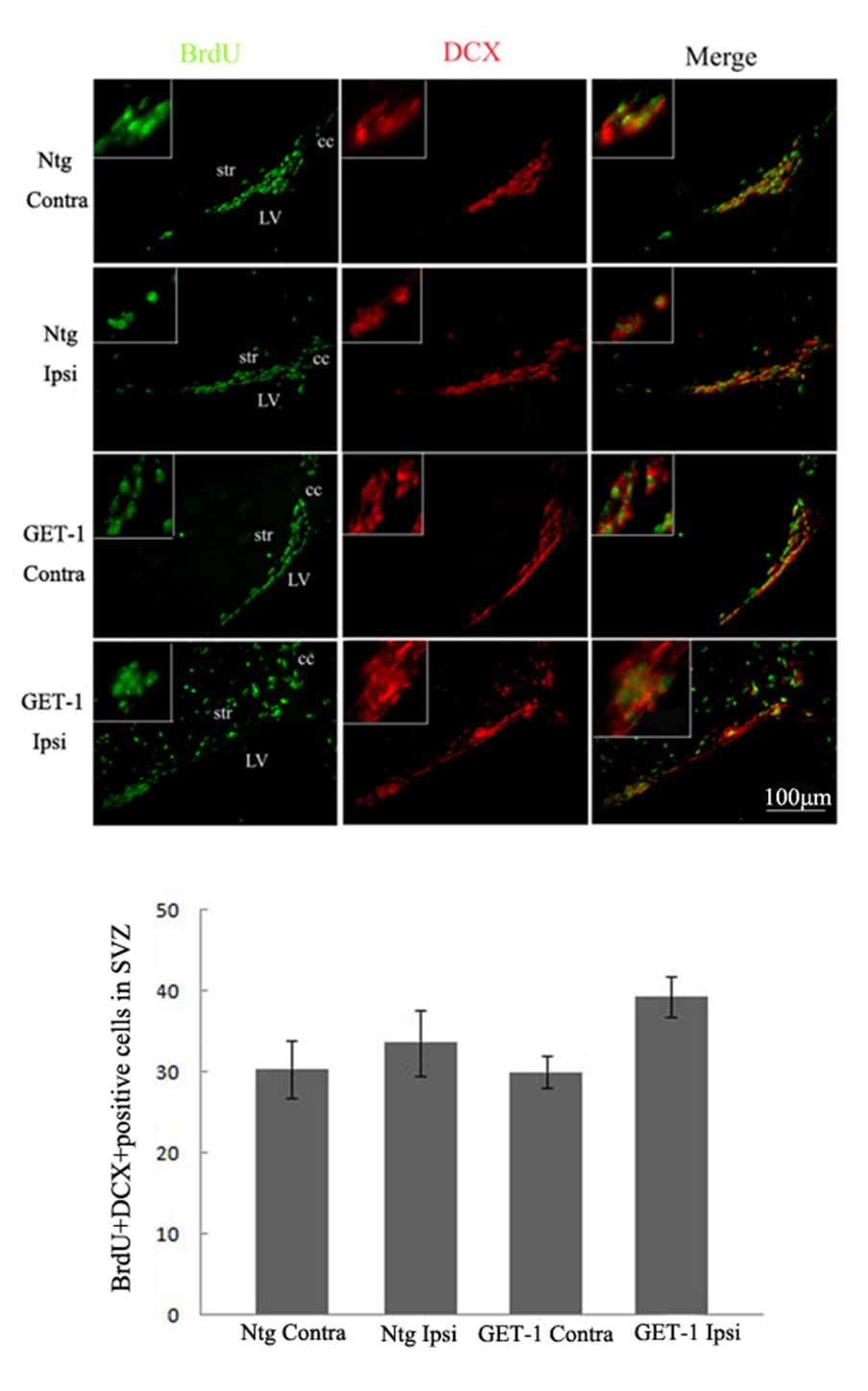

Supplement: Supplementary file 3 — Additional file 3: Figure S3. Images show immunostaining for BrdU and DCX in SVZ cells of Ntg and GET-1 mice 28 days post tMCAO. GET-1 mice did not affect SVZ cell migration in the ischemic brain after tMCAO. Neuroblast migration from the SVZ through the CC to the peri-infarct cortex BrdU+DCX+(neuroblast) double-immunostaining and area quantification in the dorsolateral ventricle (DL) area at day 7 after tMCAO in the contralateral and ipslateral side of Ntg and GET-1 mice. Scale bar=100 μm; Str=striatum; SVZ=subventricular zone; Contra=contralateral side of ischemia brain; Ips= ipsilateral side of ischemia brain. CC= Corpus callosum. [file 12974_2019_1597_MOESM3_ESM.tif]

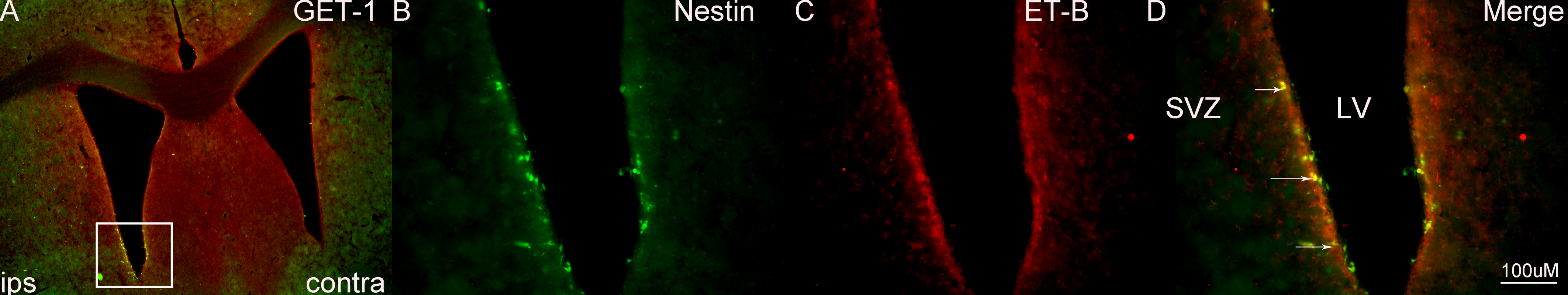

Supplement: Supplementary file 4 — Additional file 4: Figure S4. Images show immunostaining for ET-B+Nestin+ in SVZ cells of GET-1 mice 7 days post tMCAO. LV=lateral ventricle; SVZ=subventricular zone; Contra=contralateral side of the ishchemic brain; ips= ipsilateral side of the ischemic brain. Scale bar=100 μm. [file 12974_2019_1597_MOESM4_ESM.tif]

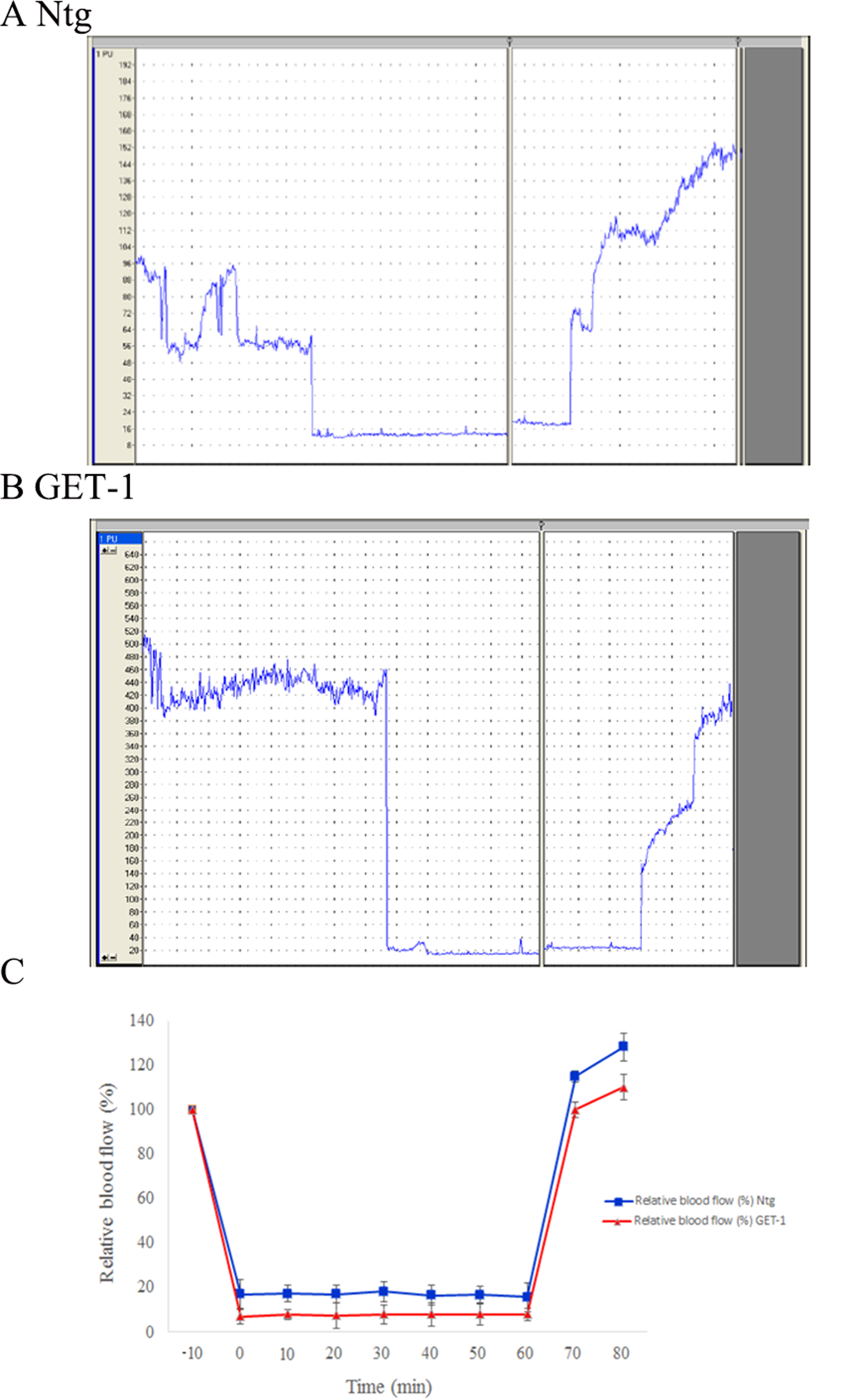

Supplement: Supplementary file 5 — Additional file 5: Figure S5. Representative read out of cerebral blood flow as monitored during tMCAO. [file 12974_2019_1597_MOESM5_ESM.tif]
